# Evaluation of the usefulness of non-invasive serum haemoglobin measurement in a perioperative setting in a prospective observational study

**DOI:** 10.1038/s41598-022-13285-z

**Published:** 2022-05-31

**Authors:** Gabriel Honnef, Daniel Auinger, Michael Eichinger, Michael Eichlseder, Philipp G. H. Metnitz, Martin Rief, Paul Zajic, Philipp Zoidl, Helmar Bornemann-Cimenti

**Affiliations:** Division of General Anaesthesiology, Emergency and Intensive Care Medicine, Medical University of Graz, Auenbruggerplatz 5, 8036 Graz, Austria

**Keywords:** Anaemia, Laboratory techniques and procedures

## Abstract

Patient Blood Management (PBM) programmes seek to reduce the number of missed anaemic patients in the run-up to surgery. The aim of this study was to evaluate the usefulness of haemoglobin (Hb) measured non-invasively (SpHb) in preoperative screening for anaemia. We conducted a prospective observational study in a preoperative clinic. Adult patients undergoing examination for surgery who had their Hb measured by laboratory means also had their Hb measured non-invasively by a trained health care provider. 1216 patients were recruited. A total of 109 (9.3%) patients (53 men and 56 women) was found to be anaemic by standard laboratory Hb measurement. Sensitivity for SpHb to detect anaemic patients was 0.50 (95% CI 0.37–0.63) in women and 0.30 (95% CI 0.18–0.43) in men. Specificity was 0.97 (95% CI 0.95–0.98) in men and 0.93 (95% CI 0.84–1.0) in women. The rate of correctly classified patients was 84.7% for men and 89.4% for women. Positive predictive value for SpHb was 0.50 (95% CI 0.35–0.65) in men and 0.40 (95% CI 0.31–0.50) in women; negative predictive value was 0.93 (95% CI 0.92–0.94) in men and 0.95 (95% CI 0.94–0.96) in women. We conclude that due to low sensitivity, SpHb is poorly suitable for detecting preoperative anaemia in both sexes under standard of care conditions.

## Introduction

Preoperative anaemia according to the World Health Organization (WHO) definition (women < 12 g dl^−1^, men < 13 g dl^−1^) has been shown to be an independent risk factor for perioperative transfusion, complications, and mortality^1–3^. 90-days mortality after surgery is five times higher in patients with pre-operative anaemia than in non-anaemic patients^4^. The risk of postoperative events is increased in patients with any degree of anaemia and independent of the type of surgery. Furthermore, 30-day postoperative mortality is increased with every percentage-point decrease of haematocrit in an elderly population^2^.

To address these issues, Patient Blood Management (PBM) programs have been established. Their introduction has been shown to be associated with improved patient outcomes, fewer transfusions, and lower costs^5–8^. Hb measurement plays an important role in the implementation of PBM programs.

Standard methods of measurement require direct blood sampling and are therefore relatively invasive, costly, and time-consuming. Recommendations to minimize unwanted side-effects of testing and to reduce unnecessary testing have been proposed. These guidelines prioritize testing for anaemia in patients with higher ASA physical health status classification scores and those undergoing moderate to major surgery, because the prevalence for anaemia in ASA 1 and 2 patients is lower than in higher-risk populations^9,10^.

Non-invasive, fast, and inexpensive means of haemoglobin measurement could reduce both these unwanted aspects of testing and the number of non-recognized anaemic patients. Haemoglobin measurement techniques utilizing a finger probe (SpHb) have become available over the last few years. Different devices based on spectrophotometric estimation of haemoglobin are commercially available, both for continuous SpHb measurement and for spot-checks of SpHb.

Previous studies have compared the accuracy of these devices with central laboratory Hb (LabHb) data in a wide range of clinical settings: operating rooms, critical care units, emergency departments, and blood donor clinics^11–14^. A meta-analysis of previous studies on continuous SpHb measurements in perioperative and intensive care settings has revealed a mean difference between SpHb and central laboratory Hb of 0.10 ± 1.37 g dl^−1^ (*n* = 4425)^12^. To date however, no studies we know of have assessed the use of these devices in a preoperative setting under standard of care conditions. With reliable cut-off values, SpHb levels could potentially serve either as a replacement for LabHb testing or as a pre-test for patients who would normally not receive a complete preoperative lab test, to further minimize the risks of undetected preoperative anaemia. We thus hypothesize that SpHb measurement is useful in preoperative screening for anaemia.

## Methods

### Study design and patient population

This was a single-centre, prospective observational study conducted in the preoperative clinic of the Department of General Anaesthesiology at the Medical University of Graz, Austria. Consecutive patients evaluated preoperatively between January and October of 2020 at the Department of Anaesthesiology and Intensive Care Medicine at the University Medical Centre, Graz, for plastic, orthopaedic, urological, general, and gynaecological surgery were included. This article was written using the STARD 2015 guidelines.

### Ethics approval and consent to participate

The study was registered at clinicaltrials.gov (NCT04391517, 18/05/2020). Approval by the ethics committee of the Medical University of Graz (IRB00002556, chairperson Prof. Josef Haas) was given on the 5th of December 2019 (decision number 31-482 ex 18/19). Patients undergoing preoperative evaluation by an anaesthesiologist were informed about the aim, intervention, and protocol of this study ahead of preoperative assessment and asked to sign a consent form. Patients were free to withdraw their consent for use of their data at any given time-point, without specification of reason in accordance with Austrian and European legislation. Informed consent was obtained from all included patients.

### Inclusion and exclusion criteria

We included male and female patients aged 18 years or above, preparing for elective surgery and willing to participate. The criteria for exclusion were thus defined as any of the following: age < 18 years, emergency surgery, no central laboratory Hb test, and patient refusal.

### Measurements and data management

Haemoglobin measurements were performed according to the standard operating procedure of our preoperative clinics. Laboratory tests for haemoglobin levels were therefore performed as part of clinical routine, except for patients in whom the assessing clinicians explicitly did not consider these investigations necessary. Anaemia was defined as a LabHb value < 12 g dl^−1^ in women and < 13 g dl^−1^ in men according to the World Health Organization (WHO)^6^.

LabHb values were documented either based on results of blood collection and laboratory analysis conducted during preoperative evaluation using a Sysmex XN-1000 (Sysmex K.K., Kobe, Japan) or from blood samples drawn no more than one month before preoperative evaluation, if the patient was in a stable condition (i.e., no active bleeding, no haematological disease, etc.), as is standard procedure at our clinics. Patients without a suitable laboratory Hb measurement were not included in the study population.

Non-invasive Hb measurements were performed additionally in patients with available LabHb values. Rad-67™ Spot-check Pulse CO-Oximeters® (Masimo corporation, Irvine, CA, USA), finger-probe devices based on multi-wavelength co-oximetry for the spectrophotometric estimation of haemoglobin (SpHb), were used according to manufacturer specifications. Trained health care providers placed the sensor on one of patients’ digits during preoperative evaluation in a sitting position. After thirty to sixty seconds, SpHb values were obtained. If the device detected low signal stability or perfusion, the other digit was used. SpHb values were recorded in our clinics’ documentation system.

### Sample-size calculation

Sample size was calculated based on a two-sided confidence interval with a confidence level 1-α of 95%, an expected proportion of 0.85 and a distance of proportion to limit of 0.05. With an expected prevalence of preoperative anaemia of 14.1%^1^, required sample size was calculated to be 1392.

### Statistical analysis

Patient characteristics were described with mean and standard deviation (SD) or as numbers and percentages (%), as appropriate. To assess the reliability of SpHb measurement for the identification of anaemic patients as defined by the WHO, sensitivity and specificity of the measurements were calculated with LabHb values as the gold standard. Differences between measurements were described according to Bland and Altman, including the calculation of mean deviation and limits of agreement (= mean deviation ± 2 × standard deviation)^15^. Precision was described with mean, the differences of measurements, and the limits of agreements.

Subgroup analyses were performed (a) in patients with moderate to severe anaemia (< 11 g dl^−1^)^14^ only, since it was shown that 30-day postoperative mortality is associated with the severity of preoperative anaemia^2^, and (b) in patients with an ASA score < 3, as these patients might not necessarily have their haemoglobin levels measured according to recommendation and anaemia could thus remain undetected.

Because of the differences in accuracy of SpHb measurement for men and women described elsewhere^14^, we primarily analysed our data stratified by sex.

Data were analysed using SAS v 9.4 (SAS Institute, North Carolina, USA) and SPSS v 26 (SPSS Inc., Stanford, USA).

### Approval for human experiments

The study was registered at clinicaltrials.gov (NCT04391517). Approval by the ethics committee of the Medical University of Graz (IRB00002556, chairperson Prof. Josef Haas) was given on the 5th of December 2019 (decision number 31–482 ex 18/19).

### Consent to participate/consent to publish

Patients undergoing preoperative evaluation by an anaesthesiologist were informed about the aim, intervention, and protocol of this study ahead of preoperative assessment. Informed consent was then obtained from all subjects. Patients were free to withdraw their consent for use of their data at any given time-point, without specification of reason in accordance with Austrian and European legislation.

## Results

**Figure 1:**
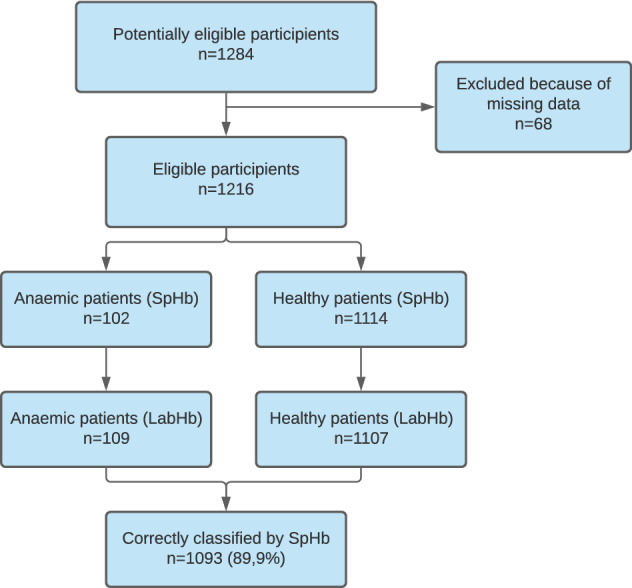
Study flow chart.

A total of 1284 patients were initially recruited from our pre-aesthetic clinics. 68 patients were excluded because of missing data (Fig. 1). Characteristics of the 1216 patients analysed are presented in Table 1. Of these, 660 (54%) were female and 556 (46%) were male. Most patients were classified according to the ASA risk categories as one and two (n = 889, 74%). (Table 1). Mean LabHb was 14.1 ± 1.5 g dl^−1^ overall, 14.9 ± 1.3 g dl^−1^ in men, and 13.4 ± 1.3 g dl^−1^ in women. A total of 109 (9%) patients were found to be anaemic with standard laboratory Hb measurement; prevalence of anaemia was similar in both sexes [n = 53 (49%) in men, n = 56 (51%) in women]. Mean SpHb was 14.2 ± 1.4 g dl^−1^ overall, 14.7 ± 1.4 g dl^−1^ in men and 13.8 ± 1.5 g dl^−1^ in women. Sensitivity of SpHb to detect anaemic patients was 0.50 (95% CI 0.37–0.63) in women and 0.30 (95% CI 0.18–0.43) in men; specificity was 0.93 (95% CI 0.84–1.0) in women and 0.97 (95% CI 0.95–0.98) in men (Table 2).

**Table 1:** Patient characteristics and haemoglobin measurements.

	Overall	Men	Women
Patients	1 216	556 (46%)	660 (54%)
Age [years] (mean, SD)	56.0 (16.3)	59.2 (15.5)	52.8 (17.1)
BMI (mean, SD)	26.9 (5.9)	27.8 (5.1)	26.2 (6.6)
**Planned surgical procedure (n, %)**			
Orthopaedic surgery	357 (29%)	183 (33%)	172 (26%)
Gynaecological surgery	300 (25%)		294 (45%)
Urological surgery	266 (22%)	234 (42%)	32 (5%)
General surgery	109 (9%)	73 (13%)	44 (7%)
Plastic surgery	167 (14%)	60 (11%)	107 (16%)
Unknown	17 (1%)	6 (1%)	11 (1%)
ASA score (mean, SD)	2.1 (0.8)	2.1 (0.8)	2.1 (0.7)
**ASA classification (n, %)**			
ASA 1	250 (22%)	121 (22%)	129 (20%)
ASA 2	639 (52%)	251 (47%)	378 (57%)
ASA 3	266 (22%)	135 (24%)	131 (20%)
ASA 4	45 (4%)	30 (5%)	15 (2%)
Missing ASA	16 (1%)	7 (1%)	9 (2%)
LabHb [g dl^−1^] (mean, SD)	14.1 (1.5)	14.9 (1.3)	13.4 (1.3)
SpHb [g dl^−1^] (mean, SD)	14.2 (1.4)	14.7 (1.4)	13.8 (1.5)

**Table 2:** Diagnostic characteristics of SpHb measurement compared to with LabHb for the diagnosis of anaemia defined as Hb < 13.0 g dl^−1^ for men and Hb < 12.0 g dl^−1^ for women.

Men *n* = 556	SpHb		
	Hb < 13.0 g dl^−1^ *n* = 32	Hb ≥ 13.0 g dl^−1^ *n* = 524	
**LabHb**			
Hb < 13.0 g dl^−1^ *n* = 53	True positive *n* = 16	False negative *n* = 37	Sensitivity 30.2%
Hb ≥ 13.0 g dl^−1^ *n* = 503	False positive *n* = 16	True negative *n* = 487	Specificity 96.8%
Prevalence 9.5%	Positive predictive value 49.9%	Negative predictive value 93.0%	Accuracy 90.5%

Women *n* = 660	SpHb		
	Hb < 12.0 g dl^−1^ *n* = 70	Hb ≥ 12.0 g dl^−1^ *n* = 590	
**LabHb**			
Hb < 12.0 g dl^−1^ *n* = 56	True positive *n* = 28	False negative *n* = 28	Sensitivity 50.0%
Hb ≥ 12.0 g dl^−1^ *n* = 604	False positive *n* = 42	True negative *n* = 562	Specificity 93.1%
Prevalence 8.5%	Positive predictive value 40.0%	Negative predictive value 95.3%	Accuracy 89.4%

Positive predictive value for SpHb was 0.50 (95% CI: 0.35–0.65) in men and 0.40 (95% CI 0.31–0.50) in women; negative predictive value was 0.93 (95% CI 0.92–0.94) in men and 0.95 (95% CI 0.94–0.96) in women (Table 2). The rate of correctly classified patients was 90% for men and 89% for women.

In the subgroup of patients with an ASA score < 3 (n = 889), 7% (n = 58) of patients were found to be anemic with LabHb. Of these 58 patients 21 (36%) were detected as anemic by SpHb. Sensitivity for the SpHb device to detect anemic patients in this subgroup was 0.45 (95% CI 0.17 to 0.77) in men and 0.40 (95% CI 0.19–0.47) in women; specificity was 0.98 (95% CI 0.95–0.99) in men and 0.98 (95% CI 0.97–0.99) in women.

In the subgroup of patients with moderate to severe anaemia (n = 38) (as defined by the WHO < 11 g dl^−1^)^16^, sensitivity was 0.56 (95% CI 0.21–0.86) in men and 0.17 (95% CI 0.06–0.36) in women; specificity was 1.00 (95% CI 0.99–1.00) in men and 0.99 (95% CI 0.98–1.00) in women.

**Figure 2:**
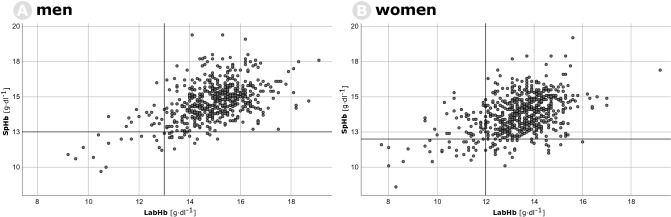
Scatterplots of LabHb (x-axis) and SpHb (y-axis) during preoperative evaluation. (**A**) Men and (**B**) women.

**Figure 3:**
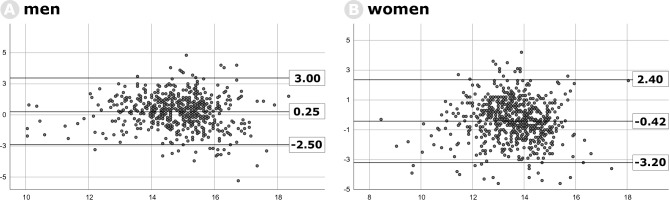
Bland–Altman-Plot for LabHb and SpHb in (**A**) men and (**B**) women; x-axis denote average between two measurements, y-axis denote differences between two measurements, horizontal lines indicate mean differences and limits of agreement between two measurements (= mean deviation ± 2 × standard deviation), respectively.

The correlation between SpHb and LabHb is shown in Fig. 2; Pearson’s correlation coefficient was of r = 0.50 for men and r = 0.45 for women. We could not find systemic variation of measurements (Fig. 3). Mean difference for men was 0.25 ± 1.3 g dl^−1^ (limits of agreement − 2.5 to 3.0) and − 0.42 ± 1.4 g dl^−1^ (limits of agreement − 3.2 to 2.4) for women.

## Discussion

In this study, we find that SpHb measurement is mostly unsuitable for detecting preoperative anaemia in both male and female patients. This is due to SpHb’s low sensitivity for detection of anaemia in this population, which results from its mediocre correlation with laboratory measurements of haemoglobin levels and spread of results when compared to laboratory measurements of haemoglobin levels as represented by wide limits of agreement. This diagnostic inadequacy made calculation of cut-off values neither feasible nor sensible.

In our study population, a high percentage of patients have been classified with ASA scores of 1 or 2 (74%). In this subgroup, we have detected 21 anaemic patients with the SpHb measurement device that would otherwise have been at risk of being missed by standard preoperative evaluation. However, more anaemic patients in this group have been missed by SpHb measurement (n = 35; 63%) then detected (n = 21; 37%).

Numerous studies have been conducted with the aim of validating SpHb measurements in different settings, with different methods and with largely different results^12–14,17–19^. In a study with comparable population (i.e., pre-aesthetic clinic patients, n = 699) and similar technology, Khalafallah et al. have found that SpHb was useful for detecting male anaemic patients, with a sensitivity of 92% in men, 57% in women and a specificity of 74% in men and 82% in women^14^. These results differ considerably from our findings. The aim of our study was to assess the usefulness of SpHb in an everyday clinical setting. Therefore, we have not used the “average of three readings” mode employed in the aforementioned study, as it is time consuming and not standard in clinical routine care. This difference in methodology might have contributed to the differences observed.

Several studies have sought to evaluate the usefulness of SpHb measurement for the screening of blood donors. Some of these studies have shown good correlations between LabHb and SpHb, but with mostly low sensitivity, the overall conclusion was that non-invasive Hb measurement did not identify anaemic patients in a healthy population with sufficient reliability^20–22^. This conclusion correlates well with our own findings.

Conversely, the usefulness of continuous SpHb measurement and trend monitoring as a tool for the detection of intraoperative blood loss has already been demonstrated. Several studies have shown that trend SpHb measurement can help estimate Hb values in various settings and reduce unnecessary red blood cell (RBC) transfusions^12,14,17,18^. A meta-analysis of continuous SpHb measurement for intraoperative evaluation of patients’ Hb and transfusion triggers has found some benefits. However, the mean difference and SD between SpHb and LabHb is high^12^.

We do not find SpHb measurement to be more suitable for men than for women. Previous studies have linked the perfusion index to the accuracy of SpHb devices^23–25^. This, in combination with the fact that the female subjects in our study were significantly younger than the male subjects and were therefore less likely to have comorbidities like arterial occlusive disease and chronic heart failure, might offer an explanation for the observed differences.

If non-invasive measurement methods, such as the one investigated in this study, prove unreliable in screening for preoperative anaemia, other means of inexpensive and non-invasive screening may alternatively be explored in the future. For example, point-of-care analysis methods using as little as a single drop of blood or even existing patient data have been demonstrated to provide sufficient information for machine-learning programs to detect disease processes including haematologic disease with promising accuracy in some settings^26–28^. Similar machine-learning applications based on known variables, information gathered during preoperative evaluation, or even simple pictures of patients’ finger nail beds could potentially provide opportunities to identify patients at risk of preoperative anaemia in the run up to surgery in the future^29^.

### Limitations

Due to the COVID-19 pandemic and the consecutive reduction of surgical schedules to increase the availability of anaesthetic and intensive care resources, we have not been able to reach our planned sample size in the study observation period. Additionally, the number of anaemic patients observed in our population is slightly lower than what we expected based on previous studies^1,30^.

The number of patients with moderate to severe anaemia in our study population is too small to make reasonable statements about the usability of SpHb measurement for the detection of moderate to severe anaemia. In our study population, the proportion of women is higher than it would be in a normally distributed population. This can be explained by the high rate of recruited patients who were preparing for gynaecological and obstetric surgery.

Because we have not altered the standard protocol for preoperative evaluation, not all patients have had their SpHb measured at the same time as their blood was sampled for LabHb. However, for those anaemic patients in whom measurements have not been performed in the same session (n = 27, 25%), mean time to central laboratory was 64.4 h (59.2), and none of these patients have been found to have unstable Hb status. Thus, the difference in sampling time is unlikely to affect findings of this study.

## Conclusion

Our data suggest that the accuracy of SpHb measurement is insufficient to replace invasive blood sampling in the preoperative evaluation of patients’ haemoglobin status. Therefore, we had to reject the hypothesis that SpHb measurement is useful in preoperative screening for anaemia (Supplementary information S1).

## Supplementary Information

Supplementary Information.

## Data Availability

Data used for analyses in this manuscript are available from the corresponding author upon reasonable request.
